# The *Legionella* effector WipB is a translocated Ser/Thr phosphatase that targets the host lysosomal nutrient sensing machinery

**DOI:** 10.1038/s41598-017-10249-6

**Published:** 2017-08-25

**Authors:** Marie S. Prevost, Nikos Pinotsis, Maud Dumoux, Richard D. Hayward, Gabriel Waksman

**Affiliations:** Institute of Structural and Molecular Biology, University College London and Birkbeck, Malet Street, London, WC1E 7HX UK

## Abstract

*Legionella pneumophila* infects human alveolar macrophages and is responsible for Legionnaire’s disease, a severe form of pneumonia. *L. pneumophila* encodes more than 300 putative effectors, which are translocated into the host cell via the Dot/Icm type IV secretion system. These effectors highjack the host’s cellular processes to allow bacterial intracellular growth and replication. Here we adopted a multidisciplinary approach to investigate WipB, a Dot/Icm effector of unknown function. The crystal structure of the N-terminal domain at 1.7 Å resolution comprising residues 25 to 344 revealed that WipB harbours a Ser/Thr phosphatase domain related to the eukaryotic phospho-protein phosphatase (PPP) family. The C-terminal domain (residues 365–524) is sufficient to pilot the effector to acidified LAMP1-positive lysosomal compartments, where WipB interacts with the v-ATPase and the associated LAMTOR1 phosphoprotein, key components of the lysosomal nutrient sensing (LYNUS) apparatus that controls the mammalian target of rapamycin (mTORC1) kinase complex at the lysosomal surface. We propose that WipB is a lysosome-targeted phosphatase that modulates cellular nutrient sensing and the control of energy metabolism during *Legionella* infection.

## Introduction

*Legionella pneumophila* is a facultative intracellular pathogen responsible for a severe form of pneumonia termed Legionnaire’s Disease^1^. Infection occurs via the inhalation of aerosols, followed by phagocytosis of the bacterium by alveolar macrophages. Within the macrophage, the bacterium avoids the endosomal pathway by modifying the early entry vesicle into a specialised replicative compartment termed the “*Legionella*-Containing Vacuole” (LCV), which progressively acquires endoplasmic reticulum-derived membranes, studded with ribosomes^2^.

During infection, *L. pneumophila* utilizes a type IV secretion (T4S) system termed the Dot/Icm secretion system to translocate effector proteins into the host cell^3–7^. Over 300 potential effectors are translocated by the Dot/Icm system. These effectors are encoded on the *L. pneumophila* chromosome and their genes comprise approximately 10% of the bacterium’s genome^8, 9^. About 20% of the effectors have been linked to a cellular pathway, and of these only half have an ascribed eukaryotic target or biochemical function^2^. Although the deletion of the T4S secretion system itself impairs intracellular growth, bacterial mutants lacking individual effectors only exhibit mild defects in intracellular replication. Indeed, several studies have demonstrated that multiple, redundant effectors target the same cellular process^2^. These factors complicate the dissection of *Legionella* effector activities.

IcmW-Interacting Protein B (WipB) is a 60 kDa *Legionella* effector of unknown function originally identified amongst a cohort of proteins that interact with the Dot/Icm T4S chaperone complex IcmS/IcmW^10^. *Legionella* deletion mutants lacking *wipB* do not exhibit a replicative defect in macrophages, unless this is combined with a second mutation in *lidA*, a gene encoding another T4S effector capable of binding Rab GTPases. *L. pneumophila wipB* ^*−*^ *lidA* ^*−*^ double mutants exhibit attenuated intracellular replication in macrophages and increased host cell death, although the significance of this remains unclear^2, 11^. Given this characteristic lack of attenuation in the single gene deletion mutant, a hallmark of functional redundancy, we adopted an alternative structural and biochemical approach to explore the properties and target/s of WipB. Our data reveal that WipB is a modular effector, with an N-terminal Ser/Thr phosphatase domain related to the eukaryotic phospho-protein phosphatase (PPP) family, and a C-terminal domain sufficient to pilot WipB to the acidified LAMP1-positive lysosomal compartments in eukaryotic cells, where WipB interacts with the v-ATPase and the associated LAMTOR1 phosphoprotein, key components of the lysosomal nutrient sensing (LYNUS) apparatus.

## Results and Discussion

### The structure of the WipB N-terminal domain reveals a phosphatase domain

**Figure 1:**
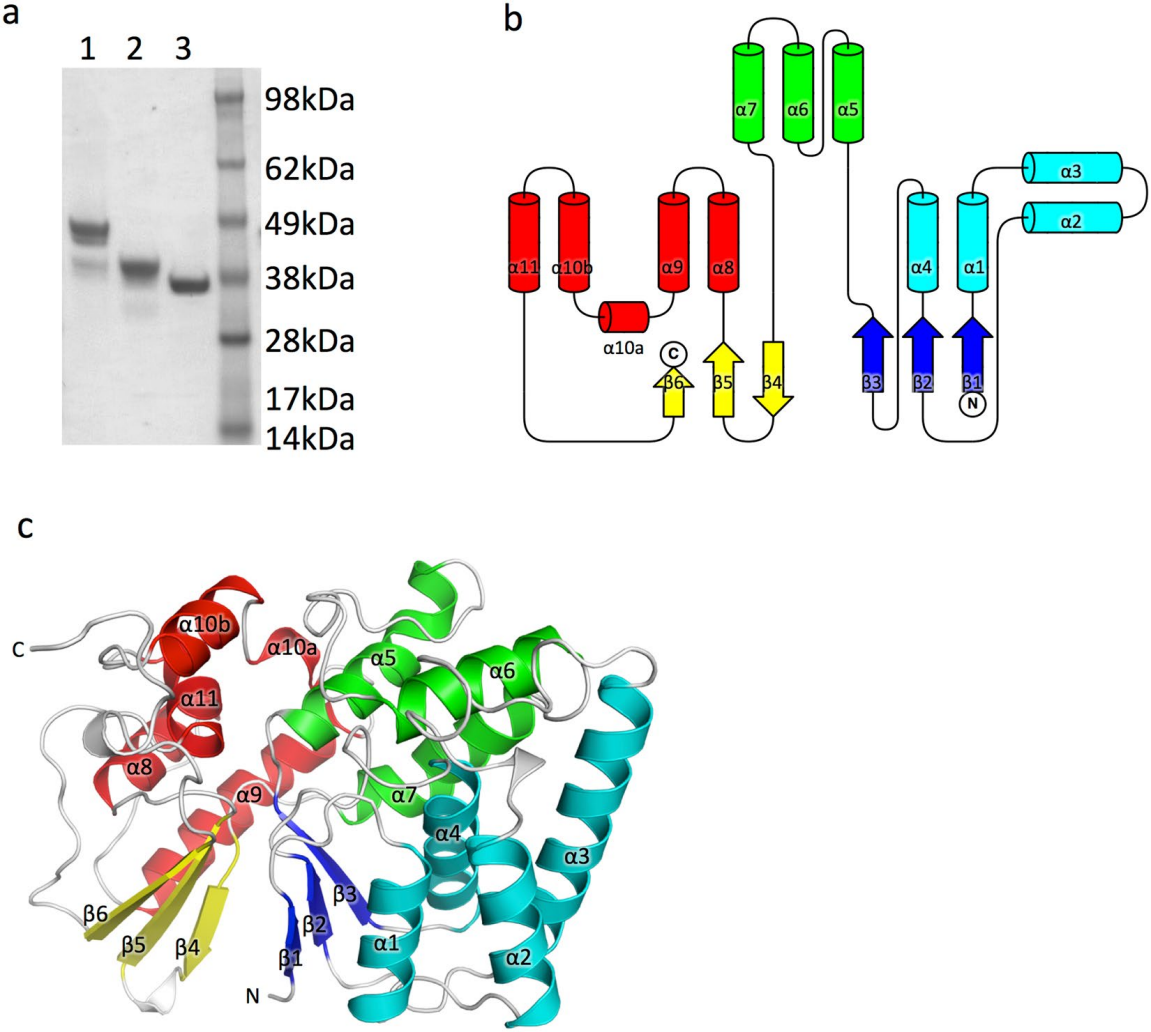
Domain structure of WipB and WipB_25-344_ structure (**a**) SDS-PAGE analysis of WipB and WipB fragments. Lane 1: full-length WipB. Lane 2: WipB_1-364_. Lane 3: WipB_25-344_. (**b**) Topology diagram of WipB_25-344_. β-strands and α-helices are shown as arrows and cylinders, respectively. The central β-sandwich is shown in blue and yellow. The flanking α-helices on the side of the blue or yellow β-sheets are shown in cyan and red, respectively. The three helices at the top are shown in green. (**c**) Three-dimensional diagram of WipB_25-344_. The structure is shown in cartoon representation with secondary structures color-coded as in panel B.

WipB was purified following over-expression in *E. coli* as described in Materials and Methods. However, we observed rapid breakdown of the protein into shorter fragments (Fig. 1a), the shortest of which was identified by mass spectrometry to contain the first 364 residues of the protein. A construct encoding this apparently stable fragment was generated and the protein expressed and purified (Fig. 1a). This fragment, WipB_1-364_, was stable but did not crystallise. Thus, the construct was further modified to exclude the regions of the N- or C-terminal sequence predicted to have no secondary structures (regions 1–24 and 345–364). The resulting fragment, WipB_25-344_, also yielded a stable protein, which in contrast to WipB_1-364_ crystallised readily. The crystals diffracted to a resolution of 1.7 Å (Table 1). The structure was solved using the Molecular Replacement method, with the recently solved structure of WipA (a protein with 34% identity at amino acid sequence level (Fig. S1)) serving as a search model^12^. Attempts to similarly produce the C-terminal domain of WipB failed, as this region of the protein appears to be unstable when expressed in bacteria. Thus, WipB appears to contain two domains, a structured N-terminal domain and a possibly less stable C-terminal domain.

**Table 1:** Data collection and refinement statistics. Information for the highest resolution shell is given in parentheses.

	WipB 25-344
**Data Collection**	
Beamline	PX13 (EMBL-PETRA III)
Wavelength (Å)	0.99999
Resolution Range (Å)	48.83–1.70 (1.74–1.70)
Space group	*P 2*_1_
Cell parameters a, b, c, β (Å, grad)	49.40, 79.10, 49.70, 98.70
Total reflections	240,903 (16,864)
Unique reflections	66,193 (4,732)
Multiplicity	3.6 (3.6)
Completeness (%)	99.3 (97.1)
Mean I/Sigma(I)	8.73 (1.21)
Wilson B-factor (Å^2^)	24.67
R_sym_ (%)	11.6 (115.1)
CC1/2	0.996 (0.480)
**Refinement**	
R_work_/R_free_ (%)	18.7/21.7
CC_work_/CC_free_	0.955/0.938
Protein atoms	5089
Solvent molecules	484
B-factor (Å^2^)	22.10
Protein	60.91
Solvent	56.39
**Ramachandran Plot**	
Favored (%)	96.15
Allowed (%)	3.69
Outliers (%)	0.16
Clash score	5.34
**Rmsd**	
Bonds (Å)	0.007
Angles (grad)	0.880
PDB code	5NNY

WipB_25-344_ crystallizes as a dimer in the asymmetric unit. While the two chains superpose very well (root-mean-square deviation (RMSD) in Cα atoms of 0.5 Å), chain A exhibits overall lower B-factors (Fig. S2a). In chain A, density for residues 332–344 is missing while the same region is resolved in chain B (Fig. S2b), This region in chain B interacts with chain A, probably an artefact of crystal packing as WipB_25-344_ is monomeric in solution.

WipB_25-344_ forms a compact structure consisting of a central β-sandwich and 11 α-helices, 4 α-helices on each side of the β-sandwich (in cyan (α1-4) and red (α8-11), respectively, in Fig. 1b,c) with three more α-helices (α5-7) capping the structure (in green in Fig. 1b,c). The structure can be further subdivided in an N-terminal lobe consisting of α1-4 and β1-3 (cyan and blue in Fig. 1b,c) on the one hand and a C-terminal lobe consisting of α8-11 and β4-6 (in red and yellow in Fig. 1b,c) on the other hand. The structure of WipB_25-344_ is very similar to that of WipA_24-435_ determined previously (the structures align with a root-mean-square deviation in Cα atoms of 1.9 Å over 279 WipB residues; Fig. S2c). However, WipA differs from WipB in including a very large α-helical hairpin (Fig. S2c).

**Figure 2:**
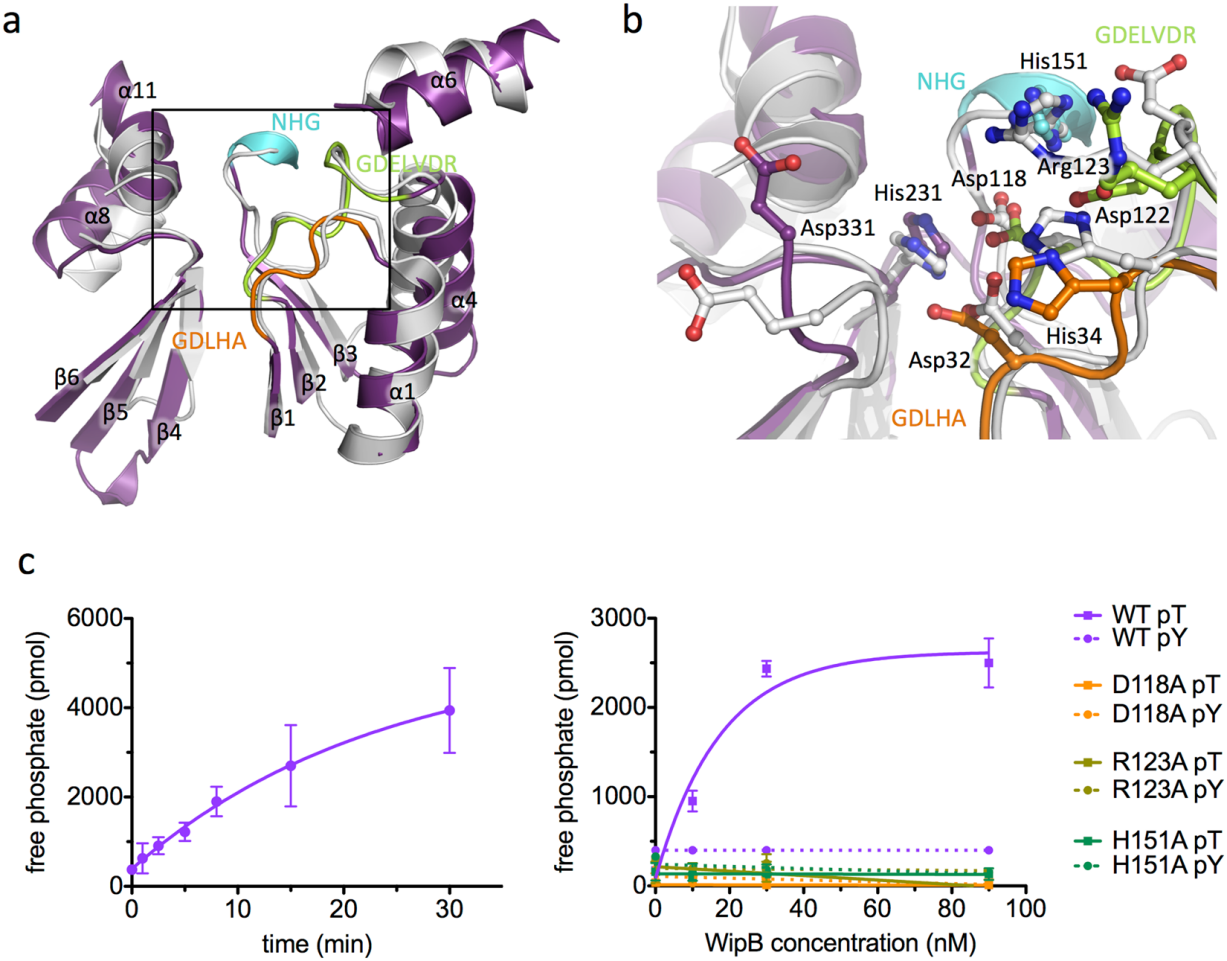
WipB is related to the PPP phosphatases (**a**) Structure-based alignment of the core part of the structure conserved between the human phosphatase PP2B (grey, pdb code 5C1V) and WipB (purple). Both structures are in cartoon representation. The three phosphatase motifs, GDLHA, GDELVDR, and NHG, are mapped onto the structure and color-coded in orange, green and blue, respectively. Orientation is the same than in Fig. 1c. (**b**) Active site of WipB and PP2B. The two proteins are shown as in panel A in the same orientation. Residues of the active site of PP2B conserved in WipB are shown in ball-and-stick representation, with oxygen and nitrogen atoms color-coded in blue and red, respectively. For residues in the three phosphatase motifs GDLHA, GDELVDR, and NHG, carbon atoms are color-coded in orange, green or blue, respectively. (**c**) Activity assay of WipB_1-364_. Left panel: release of free phosphate over time from the RRA(pT)VA peptide incubated at 37 °C with 30 nM of WipB_1-364_. Right panel: phosphate release measurements using increasing concentrations of WipB_1-364_ wild-type or mutant incubated for 2 minutes at 37 °C with either the RRA(pT)VA (solid line) or the END(pY)INASL (dashed line) peptide. Results with the wild-type protein establish that WipB is a Ser/Thr protein phosphatase while results obtained with the mutants ascertain the catalytic role of residues conserved in the structural alignment with PPPs.

A search for similar structures using the DALI server^13^ highlighted several hydrolases among the highly scored homologous structures, including the catalytic domain of a number of serine/threonine (Ser/Thr) protein phosphatases including PP1, PP2A, PP2B, and PP5, important members of the larger phospho-protein phosphatase (PPP) family of proteins^14^ Structural superposition of WipB_25-344_ with these eukaryotic phosphatases (see Fig. S1 for sequence alignment with a number of PPPs, and Figs S2d and 2a for structural superposition with PP2B) reveals a common structural core aligning with an RMSD in Cα atoms of 2 Å. This core consists of the central β-sandwich together with the flanking helices α1, α4, and α6 on one side and α8 and α11 on the other. Importantly, these regions harbour signature motifs conserved in PPPs, notably Ser/Thr phosphatases (Fig. 2a). In the PPP family, the DxH, GDxxDR, and NHE signature motifs are strictly conserved and comprise most of the residues that form the catalytic site^14^ WipB exhibits similar motifs, namely GDLHA, GDELVDR, and NHG (Fig. 2a and S1), and these motifs cluster in one particular region of WipB_25-244_, on the loops connecting β1 and α1 (GDLHA), β2 and α4 (GDELVDR), and β3 and α5 (SNG). In this region, the side chains of Asp 32 and His 34 (in GDLHA), Asp118 and Asp122 (in GDELVDR) and of His 151 (in NHG) form a site which in PP2B and other Ser/Thr phosphatase has been shown to be the catalytic site of these enzymes (Fig. 2b). Two additional residues are located in this cluster, His 231 and Asp 331, both conserved and known to be important for activity in PP2B and other PPPs (Fig. 2b).

### WipB is a Ser/Thr phosphatase

The structural homology of WipB_25-244_ with phosphatases of the PPP family suggests that WipB might contain a Ser/Thr phosphatase activity. To test this hypothesis, we investigated the ability of the WipB N-terminal domain (residues 1-364) to catalyse dephosphorylation reactions *in vitro* using a model phosphothreonine peptide with sequence RRA(pT)VA, a peptide derived from the rat liver pyruvate kinase and routinely used to monitor Ser/Thr phosphatase activities^15^. As shown in Fig. 2c, WipB_1-364_ clearly exhibits phosphatase activity. Biochemical quantification of the initial rates at 21 °C revealed a K_m_ of 0.5 ± 0.3 mM and a V_max_ of 0.042 ± 0.02 mM.s^−1^ for the dephosphorylation reaction using 30 nM of WipB_1-364_.

Since WipB and WipA are structurally similar and WipA exhibits tyrosine protein phosphatase activity, we next tested the activity of WipB against a phosphotyrosine-containing peptide (Fig. 2c). The model peptide END(pY)INASL (a peptide derived from the T cell phosphatase sequence^16^ and generally used to assay tyrosine phosphatase activities) was used as substrate in the presence of 0, 10, 30 and 90 nM of WipB_1-364_ and the released free phosphate concentration was measured after incubation at 37 °C for 2 minutes (Fig. 2c). The same experiment was carried out using the RRA(pT)VA peptide for comparison. We observed that WipB_1-364_ is able to release phosphate from the phosphothreonine, not the phosphotyrosine, peptide. Another phosphotyrosine-containing peptide routinely used to monitor tyrosine dephosphorylation was also used, with sequence DADE(pY)LIPQQG derived from the EGFR protein^17^, and the lack of activity against phosphotyrosine-containing sequences was confirmed (data not shown).

In order to assess whether the active site identified from comparison with the structure of PP2B is responsible for the measured phosphatase activity, we expressed and purified three derivatives of WipB_1-364_ each containing an individual point mutation within the putative active site: Asp118 to Ala (WipB_1-364;D118A_; in GDLHA motif) and Arg123 to Ala (WipB_1-364;R123A_; in the GDELVDR motif) and His151 to Ala (WipB_1-364;H151A_; in the NHG motif). The three mutants exhibited a loss in phosphatase activity confirming that the catalytic domain predicted from our structure is indeed functional (Fig. 2c).

To understand the differences between WipB and WipA specificity, we compared the structure of their catalytic sites (Fig. S2e). Both are essentially similar. However, four residues differ in position: Arg185 (123 in WipA), Asp30 (31 in WipA), Asp396 (331 in WipA) and Asp310 (369 in WipA). In WipA, the first three are involved in catalysis while Arg369 of WipA was hypothesized to be involved in pTyr recognition^12^. In WipB, Arg310 (equivalent of Arg369 in WipA), is conformationally restrained away from the active site and therefore cannot assume a role in substrate recognition. We hypothesize that this is the reason why WipB is inactive against pTyr-containing peptides.

Altogether, these data demonstrate that WipB harbours a functional N-terminal phosphatase domain that can hydrolyse Ser/Thr phosphorylated peptides *in vitro* and is catalytically independent from the C-terminal domain.

### WipB locates to the lysosomal compartment and its C-terminal domain determines its cellular localisation

Having established that WipB is a Ser/Thr protein phosphatase, we next investigated the localisation of WipB when expressed in cultured mammalian cells.

Cultured HeLa cells were transiently transfected with plasmids encoding GFP fusions of WipB, either at the C-terminus (WipB-GFP) or the N-terminus (GFP-WipB). After 24 h, immunoblotting of transfected cell lysates using an anti-GFP antibody revealed the presence of a single species at the expected molecular weight of WipB-GFP and GFP-WipB (Fig. S3a), verifying that in contrast to expression in *E. coli*, the fusion proteins were not truncated or degraded, irrespective of the location of the GFP.

**Figure 3:**
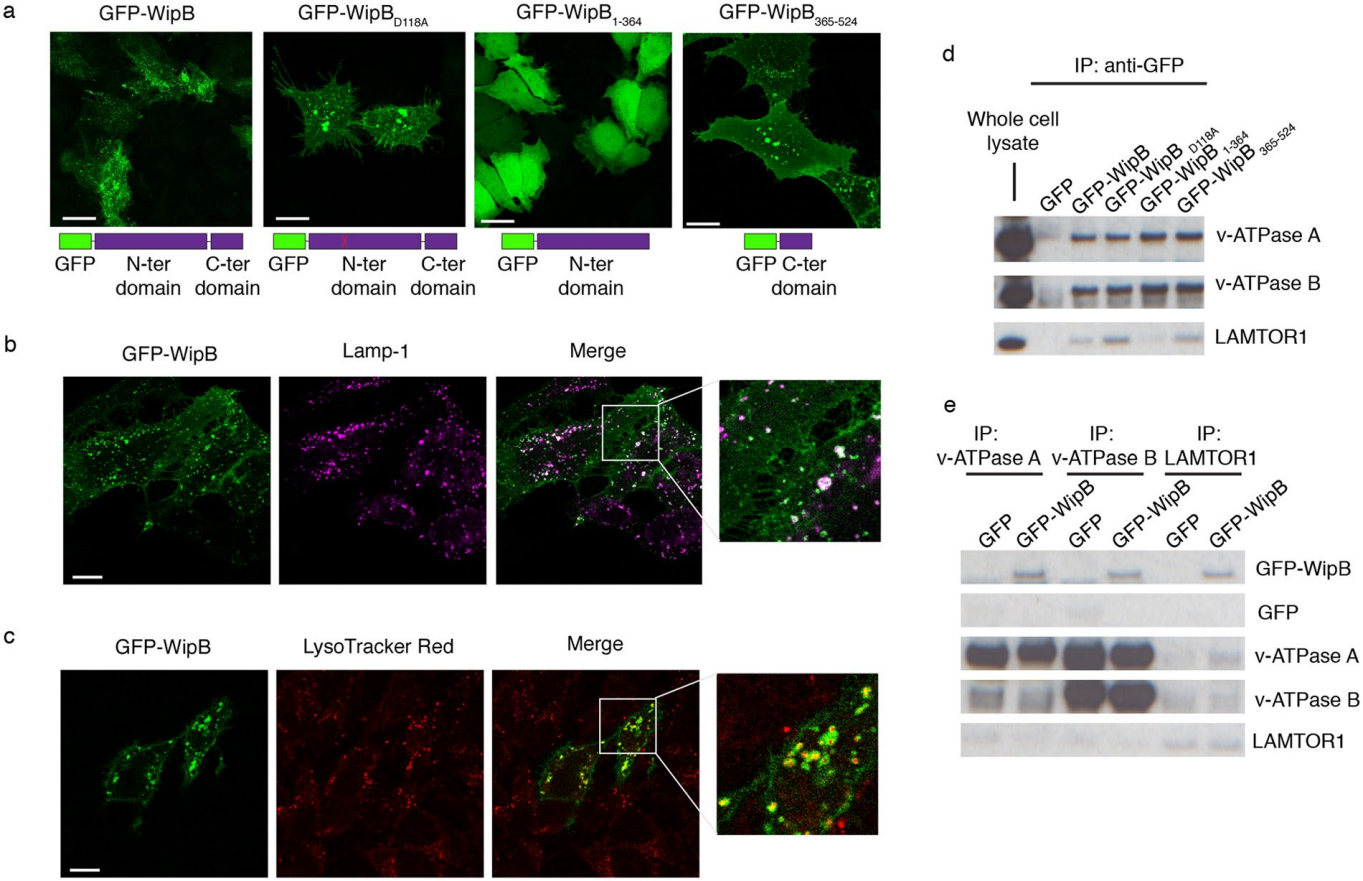
WipB is targeted to lysosomes by its C-terminal domain where it interacts with components of the lysosomal nutrient sensing system (**a**) HeLa cells expressing GFP-fusion proteins of WipB, WipB_D118A_, WipB_1-364_ and WipB_365-524_. Scale bar, 10 µm. (**b**) HeLa cells expressing GFP-WipB (green) stained using an anti-LAMP-1 antibody (Magenta) after permeabilisation and fixation. Scale bar, 10 µm. (**c**) HeLa cells expressing GFP-WipB (green) were incubated with Lysotracker (red) for 15 min before fixation. Scale bar, 10 µm. (**d**) SDS-PAGE of fractions following co-immunoprecipitation of GFP, GFP-WipB or the indicated GFP-WipB derivatives from HeLa cell lysates using anti-GFP antibody and immunoblotting with anti-v-ATPase A, -v-ATPase B or LAMTOR1 antibodies. A cropped blot is here displayed and the corresponding full-length blot is included in the supplementary information. (**e**) SDS-PAGE of fractions following co-immunoprecipitation of v-ATPase A, v-ATPase B or LAMTOR1 from lysates of HeLa cells expressing GFP or GFP-WipB and immunoblotting with anti-GFP antibody. A cropped blot is here displayed and the corresponding full-length blot is included in the supplementary information.

When transfected HeLa cells were examined using confocal microscopy, GFP-WipB and WipB-GFP predominantly formed puncta in the cytoplasm, with a minor fraction also evident at the plasma membrane (Fig. 3a for GFP-WipB and Fig. S3b for WipB-GFP). Equivalent localisation was observed when cultured retinal pigment epithelial (RPE-1) cells were transiently transfected with GFP-WipB (Fig. S3c).

To investigate whether the GFP-WipB puncta were targeted to any specific cellular compartment or whether GFP-WipB expression influenced the distribution or morphology of cellular organelles, GFP-WipB transfected cells were fixed and co-stained with markers characteristic of different subcellular compartments and organelles. GFP-WipB did not co-localise or disrupt the morphology of the Golgi apparatus (giantin), mitochondria (MitoTracker®) or the rough endoplasmic reticulum (calreticulin) (Fig. S4). However, GFP-WipB was strongly enriched around LAMP-1 positive compartments (Fig. 3b). Similarly, when transfected cells were loaded with LysoTracker® that specifically labels acidified compartments, LysoTracker-positive compartments also coincided with WipB puncta (Fig. 3c). In addition, when cells expressing GFP-WipB were loaded with TRITC-Dextran 10,000, which accumulates in endosomal compartments, partial co-localisation with the WipB puncta was also evident (Fig. S3d). Indeed, GFP-WipB could be frequently and reproducibly visualised as a defined ring of fluorescence surrounding LAMP-1 positive, LysoTracker-positive and TRITC-Dextran loaded compartments in transfected cells (Fig. 3c and S3d, zoom panels), indicative of protein recruitment. We next assessed the extent of colocalisation by calculating Manders coefficients to allow an unbiased evaluation of the degree of overlap between GFP-WipB and the various cellular markers. The resulting quantitative analysis strongly reinforced our qualitative observations, demonstrating statistically significant associations between WipB-GFP and LysoTracker, WipB-GFP and TRITC-Dextran, and most strikingly between WipB-GFP and LAMP1, but not between WipB-GFP and giantin (Fig. S3e). Correspondingly, the reciprocal analysis confirmed identical relationships when co-localisation was considered with respect to the individual cellular markers and GFP-WipB (Fig. S3e). These data reveal that the Ser/Thr phosphatase WipB is recruited to acidified LAMP1 compartments, characteristic of lysosomes.

To establish whether the Ser/Thr phosphatase activity influenced WipB localisation, cells were equivalently transfected with the catalytically dead GFP- WipB_D118A_ derivative. As with active GFP-WipB, GFP- WipB_D118A_ was stable after expression (Fig. S3a), and adopted a localisation indistinguishable from GFP-WipB (Figs 3a, S3d and S3f), demonstrating that Ser/Thr phosphatase activity is not a determinant of WipB localisation in eukaryotic cells.

To identify the region of WipB responsible for lysosomal targeting, GFP fusions of the isolated catalytic domain (GFP-WipB_1-364_) and C-terminal domain (GFP-WipB_365-524_) were equivalently transfected. Analysis of protein expression by immunoblotting of transfected cell lysates showed that each derivative was stably expressed as a fusion of the expected molecular weight (Fig. S3a). GFP-WipB_1-364_ and GFP-WipB_365-524_ were then visualised following transfection of HeLa cells using confocal microscopy (Fig. 3a). In contrast to GFP-WipB, GFP-WipB_1-364_ distributed uniformly throughout the cytoplasm, mirroring the localisation of control GFP. Strikingly, GFP-WipB_365-524_ exhibits a localization similar to GFP-WipB, demonstrating that the C-terminal domain is sufficient for lysosomal targeting.

### WipB interacts with components of the eukaryotic LYNUS apparatus

The targeting of the WipB Ser/Thr phosphatase to lysosomes by the C-terminal domain, suggested that WipB might target host phosphoproteins within the endo-lysosomal system. To identify potential host targets of WipB, we performed co-immunoprecipitation experiments with anti-GFP antibodies from transfected cell lysates, followed by mass spectrometry analysis, using HeLa cells expressing GFP-WipB, the catalytically dead derivative GFP-WipB_D118A_, or GFP alone as a control. The proteins identified under each condition were compared and purged of those interacting with GFP alone. The proteins remaining in the GFP-WipB and GFP-WipB_D118A_ datasets were strikingly enriched for lysosomal proteins with 23% of the lysosomal membrane proteome represented amongst the data^18^. Amongst the common targets, we identified lysosomal proteins present within the top 50 hits that are also predicted to contain Ser/Thr phosphorylation sites according to the UNIPROT database. This identified flotillin-1, LAMTOR1 (p18), and two subunits of the vacuolar H^+^-ATPase (v-ATPase), subunits B and d1 (Table 2). Three of these four proteins are functionally linked, as LAMTOR1 and the two v-ATPase subunits interact as part of a larger macromolecular assembly termed the lysosomal nutrient sensing (LYNUS) apparatus that controls the mammalian target of rapamycin (mTORC1) kinase complex at the lysosomal surface^19^. LAMTOR1 is a phosphoprotein that links the Ragulator complex to the lysosomal surface, which is also involved in mTOR signalling and is activated via the v-ATPase^20^. Therefore, we focussed on these three WipB interactors.

**Table 2:** Lysosomal proteins present within the 50 top-scores for both the pull-downs performed with the wild-type or D118A GFP-WipB.

Accession number	Protein	GFP-WipB^1^	GFP-WipB_D118A_^1^	GFP-WipB_1-364_	Phosphorylation site(s)^2^
O75955	Flotillin-1	+	+	−	+
Q6IAA8	Ragulator complex protein LAMTOR1	+	+	−	+
P21281	V-type proton ATPase subunit B, brain isoform	+	+	+	+
P61421	V-type proton ATPase subunit d 1	+	+	+	+

^1^Within the 50 best scores, ^2^As referenced in Uniprot.

Firstly, we verified our mass spectrometry results by immunoblotting the anti-GFP immunoprecipitation samples using antibodies specific for subunits A and B of the v-ATPase and against LAMTOR-1 (Fig. 3d and Fig. S5a). These data show that GFP-WipB and GFP-WipB_D118A_, but not GFP alone, can indeed immunoprecipitate the three target proteins.

Secondly, equivalent immunoprecipitations with N-terminal GFP-WipB_1-364_ and C-terminal GFP-WipB_365-524_ were performed (Fig. 3d and Fig. S5a). While GFP-WipB_365-524_ interacts with the two v-ATPase subunits and LAMTOR1, GFP-WipB_1-364_ only binds the v-ATPase subunits. This reveals that while both domains of WipB can engage with the v-ATPase subunits, only the C-terminal domain can interact with LAMTOR1.

Finally, we performed reverse co-immunoprecipitation using antibodies against the v-ATPase A and B subunits and LAMTOR1 (Fig. 3e and Fig. S5b). Cells were transfected with either GFP alone or GFP-WipB and lysates incubated in the presence of beads coupled to antibodies against the v-ATPase A or B subunits, or LAMTOR1. Immunoprecipitated proteins were then analysed by SDS-PAGE and Western blotting using anti-GFP antibody. As shown in Fig. 3e, all three proteins were able to pull-down GFP-WipB, but not GFP alone.

Altogether, these results show that the WipB Ser/Thr phosphatase targets key components of the host LYNUS complex on the lysosome, when expressed in mammalian cells.

## Discussion

In this study, we determined the structure and characterized the catalytic domain of the *Legionella pneumophila* effector WipB. We showed that this domain belongs to the PPP family of eukaryotic phosphatases and has a Ser/Thr phosphatase activity *in vitro* ^21^.

Ser/Thr phosphatases regulate a wide number of processes in eukaryotic cells, including targeting signalling cascade components to membrane receptors^14^. Their substrate(s) specificity often resides within their regulatory domains/subunits. Similarly, we showed that WipB has a specific subcellular localisation in HeLa cells controlled by its C-terminal domain, which targets it to lysosomes.

We demonstrated that WipB can interact with subunits of the v-ATPase and LAMTOR1 when expressed in cultured cells. The v-ATPase is a transmembrane complex that controls the acidification of cellular organelles, comprised of 17 subunits^22^, which harbour several phosphorylation sites that regulate pump function^23^. *Legionella* may modulate the function of the v-ATPase since part of the LCV membrane is derived from the lysosomal compartment^24, 25^, yet the lumen remains at neutral pH^26^. Indeed, the *Legionella* effector SidK binds the v-ATPase regulatory subunit VatA, resulting in the inhibition of the complex and organelle acidification^26^. As with *wipB* mutants, a *sidK* mutant did not exhibit defects in intracellular replication^26^. Consequently, it is possible that redundant functions of SidK and WipB converge to repress the activity of the host v-ATPase through direct binding, promoting bacterial growth.

Recently, mTOR-driven metabolic reprogramming was found to be important for intracellular replication by *Legionella* ^27^. An unknown T4S effector/s was proposed to activate mTOR to promote LCV expansion, a process that required a functional Dot/Icm secretion system and the IcmS chaperone, in addition to host phosphatidylinositol-3-kinase. Lysosomes play an important role in coordinating nutrient sensing and signalling pathways involved in cell metabolism and growth. LAMTOR1 is a key phosphoprotein of this system^20^, directing the nucleation of the multi-component LYNUS apparatus including mTOR and v-ATPase on the lysosomal surface^19^, although its mode of regulation are not yet clearly understood. Although further investigation is required, it is tempting to speculate that WipB is a T4S effector involved in the metabolic reprogramming of *Legionella*-infected cells.

Using transposon site hybridization (TraSH), several “functional groups” of effectors were proposed to concomitantly act on cellular pathways: while individual effector deletion has no effect, their combined deletion altered *L. pneumophila* growth in host cells^11^. While single deletion of *wipB* and *lidA* are phenotypically silent, a *wipB*/*lidA* double mutant exhibited defects in the stability of the LCV. During infection, LidA promotes the recruitment of ER-derived vesicles to the LCV through its binding to the AMPylated form of the Rab1 GTPase^28^. In this manner, LidA contributes to replication-niche formation, essential for *L. pneumophila* to gather the resources it needs for cell division. On the other hand, escaping the host defence mechanisms by repressing the lysosomal pathway, a process WipB could be involved in, appears crucial to bacterial survival. Thus, impairing both LidA and WipB function would alter two main complementary virulence strategies, eventually leading to intracellular growth defects.

A recent genetic study conducted on the genomes of 41 *Legionella* species remarkably showed that effector repertories share some conserved domains within and between species^29^. Those domains, catalytic, binding or uncharacterized domains, are shuffled to generate effector pools, which consist of nearly 6000 effectors in the whole sequenced genus. Using the *L. pneumophila* effectors, the authors define 608 orthologue groups of proteins sharing one or several conserved domains, representing 80% of the 6000 putative proteins. One orthologue group is based on the WipB sequence (group number LOG_00109), which according to their findings, could have orthologues in 32 out of the 41 sequenced *Legionella* species. In addition, a search of homologues to the WipB catalytic domain using BLAST^30^ also identifies hypothetical proteins effectors in *Coxiella* and *Fluoribacter* species that could then share the same enzymatic activity. Thus our findings on *L. pneumophila* WipB might have implications for understanding a whole family of effectors from the entire *Legionella* genus.

*L. pneumophila* secretes a wide range of protein effectors during the infection that leads to Legionnaire’s disease. Assigning the functions of these effectors is often complex given the high redundancy they exhibit. Here we demonstrate how combining structural biology and cellular biology is a powerful alternative route to identifying the role and targets of *Legionella* effectors.

## Methods

### Cloning

The WipB DNA (AAU28775) encoding the wild type protein (Q5ZS02_LEGPH, lpg2718) was cloned in a modified pETM14 vector (EMBL) or in empty pEGFP-C2 and -N2 vectors using a PCR-based in-fusion HD cloning system (Clontech Laboratories). For the pETM14-derived constructs, the expression cassette contained an N-terminal deca-histidine tag followed by a 3 C protease cleavage site. Site directed mutagenesis was performed using standard molecular biology protocols.

### Expression and purification of WipB_25-344_ and WipB_1-364_

All recombinant proteins were over-expressed in C43(DE3) bacterial strains. The cells were harvested by centrifugation (6000 g, 15 min) and resuspended in a lysis buffer containing 25 mM Tris-HCl pH 7.5, 0.3 M NaCl, 5 mM β-mercaptoethanol (βME), 10 mM imidazole, 5% glycerol, a tablet of protease inhibitors (Complete, EDTA-free by Roche) and 0.25 mg/ml lysozyme. Cells were lysed in an EmulsiFlex-C3 homogeniser (Avestin) and the crude extract was centrifuged at 50,000 g for 45 min. The supernatant was loaded onto a 5 ml HisTrap column (GE Healthcare) equilibrated with the lysis buffer, connected on an AKTA purifier (GE Healthcare). Washing steps were performed with extended volumes of lysis buffer though the column as well high salt buffer (25 mM Tris-HCl pH 7.5, 1 M NaCl, 5 mM βME, 10 mM imidazole, 5% glycerol). The protein was eluted using an imidazole gradient (Elution buffer: 25 mM Tris-HCl pH 7.5, 150 mM NaCl, 5 mM βME, 0.6 M imidazole, 5% glycerol). The eluted protein was dialyzed overnight against buffer A (25 mM Tris-HCl pH 7.5, 5% glycerol) in presence of 3C-protease fused to GST to a molar ratio protein/protease of 50:1. The cleaved protein was collected in the flowthrough of two 1 ml HisTrap and GST-Trap columns (GE Healthcare) connected in sequence to remove uncleaved proteins and the protease. It was then loaded on a resource Q column (GE healthcare) and eluted with a gradient of NaCl at a concentration of about 0.1 M NaCl. The eluted protein was further concentrated and loaded to a superdex200 16/60 column (GE healthcare) equilibrated in 25 mM Tris-HCl pH 7.5, 0.15 M NaCl, and 5% glycerol.

### Phosphatase activity assay

Assays were conducted on purified protein after His-tag removal using the Tyrosine and Ser/Thr Phosphatase Assay Systems kits (Promega). Briefly, the protein was diluted in its purification buffer containing 1 mM of MnCl_2_ and incubated at either 21 °C or 37 °C with the provided peptide before stopping the reaction with a buffer containing molybdate and malachite green to measure free phosphate at 600 nm in a 96-well microplate. One phosphothreonine-containing peptide (sequence RRA(pT)VA) and two phosphotyrosine-containing peptides (END(pY)INASL and DADE(pY)LIPQQG) were provided in the kits and were used as per manufacturer’s instructions.

### Crystallization, data collection and processing

Initial crystallization screens were performed using the sitting-drop vapor-diffusion technique, by mixing equal volumes (0.2 μl) of protein solution (13 mg/ml) at 16 °C. Crystals in the shape of thin needles appeared after 3-4 days reaching a maximum length of 0.1-0.2 mm against a reservoir solution containing 0.23 M ammonium citrate dibasic and 22% PEG3000. Before data collection, harvested crystals were immersed in a solution containing the precipitant mixture and 10% MDP and cryo-cooled in liquid nitrogen. All data sets were collected at 100 K. Crystals of the WipB_25-344_ were collected at the PetraIII P13 beam-line (EMBL-Hamburg/DESY P13, Germany). The data set was indexed, processed and scaled using the XDS package^31^, (Table 1).

### Structure determination and refinement

The WipB_25-344_ crystals belonged to the *P* 2_1_ space group with a solvent content of 43.12% corresponding to two molecules per asymmetric unit (AU). The structure was determined by molecular replacement using MOLREP^32^ and the WipA structure (PDB Code 5N72) containing only the α-helices and β-strands of the phosphatase domain as search model. The coordinates were further improved by maximum-likelihood and TLS refinement using the PHENIX suite^33^ and manual improvements of the model using COOT^34^. The final model converged to a final R_work_/R_free_ of 0.187/0.217 at a resolution of 1.70 Å. The WipB_25-344_ model covers the WipB amino-acid sequence 24-331 (chain A) and 25–344 (chain B) and contains in addition 484 water molecules.

### Antibodies and cellular biology reagents

CHIP grade rabbit anti-GFP (Invitrogen) was used for co-immunoprecipitation and mouse anti-GFP (clontech) for Western-blotting. Rabbit anti-vATPase A (Abcam), anti-vATPase B (Abcam) or anti-LAMTOR1 (Sigma) were used for co-immunoprecipitation and Western blotting. Anti-Sec. 61b (Millipore) was used as a loading control in Western-blotting. HRP-coupled anti-mouse and anti-rabbit (Invitrogen) and TrueBlot anti-rabbit (Rockland) were used for Western-blotting and detected using ECL kit (GE Healthcare). For cell labelling, we used anti-LAMP1 (rabbit, NEB), anti-Giantin (rabbit, Covance), anti-LaminA/C (mouse, Santa Cruz), and MitoTracker, LysoTracker Red, Dextran TRITC 10,000 and Transferrin647 were purchased from Thermofisher.

### Cell culture, transfection, labelling and microscopy

Hela cells were routinely cultured, transfected and immunolabeled as previously described^35^. For *in vivo* labeling, cells were incubated at 37 °C for 15 minutes with Dextran TITRC 10,000 at 1 mg/ml, MitoTracker at 250 nM, Transferrin-Alexa647 at 0.025 mg/ml or LysoTracker Red at 50 nM in DMEM then washed in HBSS. Transferrin-labeled cells were stripped in stripping buffer (150 mM NaCl, 100 mM Glycine, 5 mM KCl, 1 mM CaCl2, pH4.5).

Cells were imaged using a confocal microscope (TCS Sp5 AOBS; Leica) with an oil-immersion objective (63 × , 1.4 NA; Leica) as previously described^35^. Images were analyzed using Fiji^36^.

### Mass spectrometry analysis

HeLa cells were sonicated 24 h after transfection in PBS containing protease inhibitors. Lysates were incubated for 30 min at 30 °C with beads coupled to anti-GFP antibodies equilibrated in PBS Tween 0.02%(v/v). After washing, beads were processed in mass spectrometry as described previously^35^.

### Co-immunoprecipitation

Dynabeads protein G (Thermofisher) were incubated with anti-GFP, anti-vATPase A, anti-vATPase B or anti-LAMTOR1 antibodies in a buffer PBS Tween 0.02% (v/v) 10% BSA (w/v) for 10 min. Cell lysates were incubated with the washed beads for 1 h in the presence of 5% BSA to reduce non-specific interactions. After washing, proteins were eluted with 75 mM Tris HCl pH 7.4, 0.5 M EDTA, 0.05% SDS and analyzed by western blotting.

## Electronic supplementary material

Supplementary information
