# Supplementary material for: The Legionella effector WipB is a translocated Ser/Thr phosphatase that targets the host lysosomal nutrient sensing machinery
Source: Sci Rep. 2017 Aug 25;7:9450. doi: 10.1038/s41598-017-10249-6 (PMC5572681; doi:10.1038/s41598-017-10249-6)

Supplementary Information

The *Legionella* effector WipB is a translocated Ser/Thr phosphatase that targets the host lysosomal nutrient sensing machinery

Marie S. Prevost[&], Nikos Pinotsis[&,*], Maud Dumoux, Richard D. Hayward* and Gabriel Waksman*

Institute of Structural and Molecular Biology, University College London and Birkbeck, Malet Street, London WC1E 7HX, UK

[&]: co-first authors

*: authors for correspondence

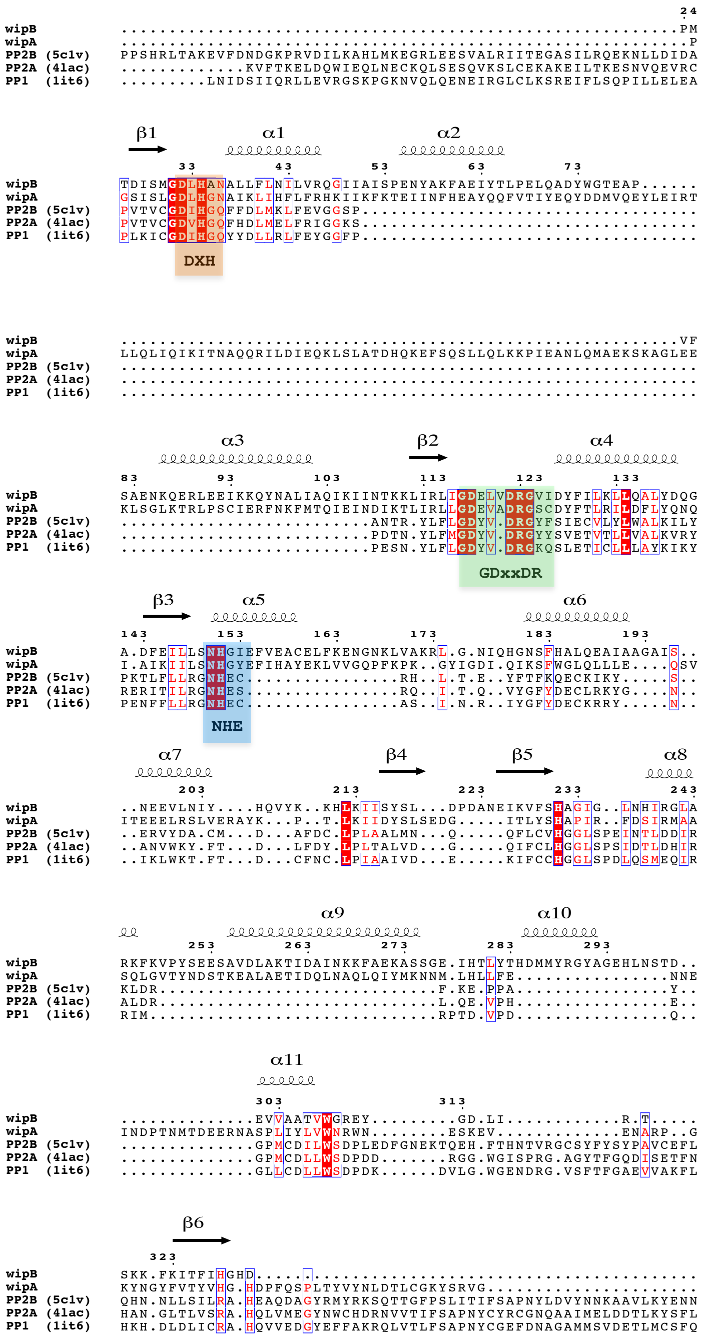

Supplementary Figure 1.

Structure-based sequence alignment of the $WipB_{25\text{-}344}$ with WipA (PDB entry code 5N6X), PP2B (PDB entry code 5C1V), PP2A (PDB entry code 2IAE) and PP1 (PDB entry code 5INB). Similar structures search was performed using the Dali server [1], selected top-scores aligned with PDBeFold [2] and the resulting alignment was rendered with ESPript [3]. The WipB secondary structure elements are shown above the aligned sequences. Conserved phosphatase motifs are labeled DXH, GDxxDR, and NHE, and color-coded orange, green and blue, respectively.

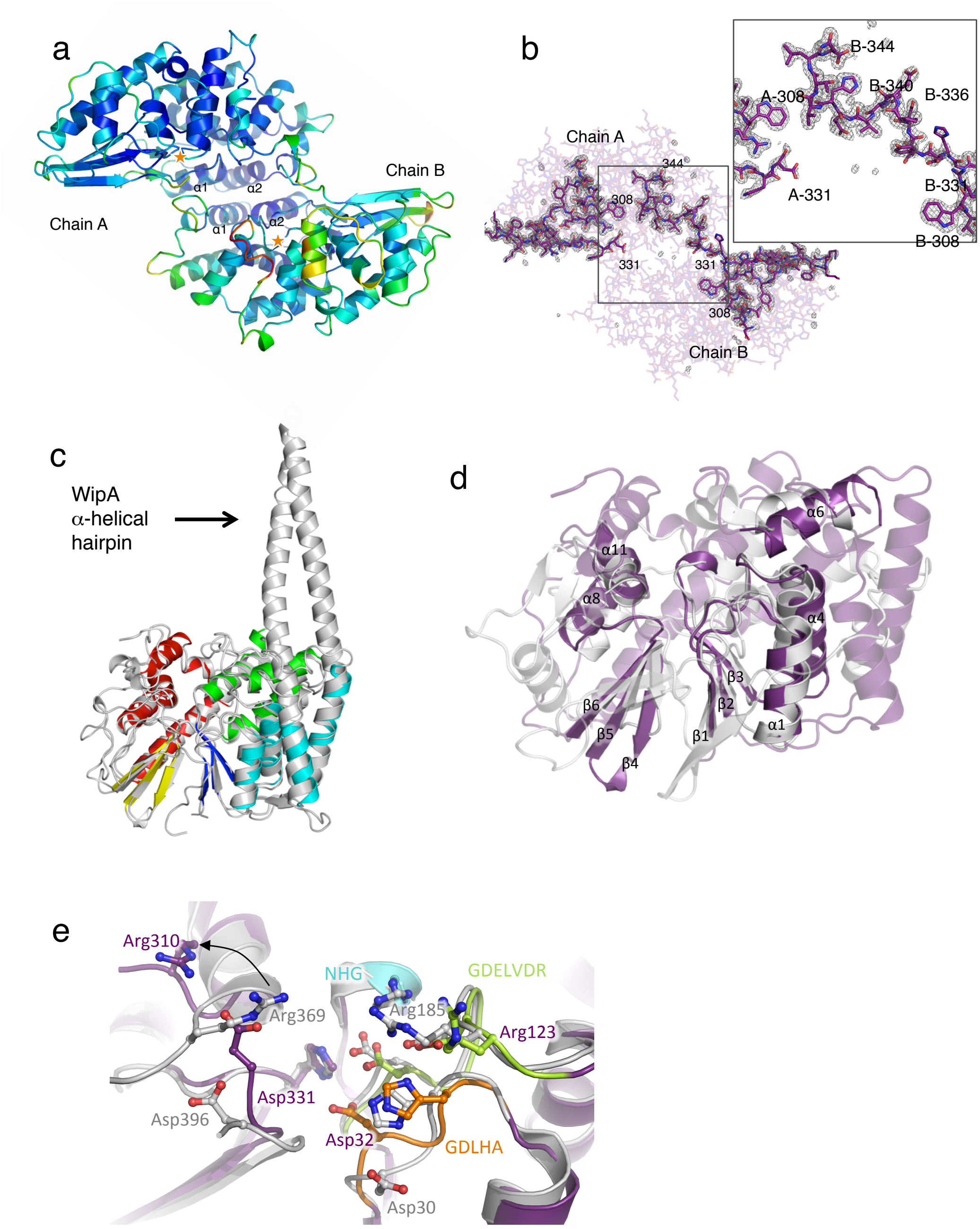

Supplementary Figure 2: Structure of the asymmetric unit of WipB$_{25\text{-}344}$ and comparison with WipA.

a: The two chains seen in the asymmetric unit are coloured according to their Cα atoms B-factors.

b: The two chains are represented in sticks, with the C-terminal part (280-344) in full colour. The electron density (calculated 2Fo-Fc coefficients and phases derived from the finally refined model) map is shown for the 280-344 residues (contoured at 1 sigma) highlighting the lack of density for the end of chain A. A close-up view of the region where the chain ends is shown on the right. Some residues are labelled to help orientation.

c: Superposition between WipA and WipB. Both structures are shown in cartoon representation. Color-coding for WipB is as in Fig. 1C, while WipA is in grey.
d: Structure-based sequence alignment of WipB and the human PPP phosphatase PP2B (PDB entry code 5C1V). Both are in semi-transparent cartoon representation, except for the conserved common parts of both structures that are not transparent. PP2B and WipB are in grey and purple, respectively. The orientation of the protein is the same as in Fig. 1C.
e: Superposition between the catalytic sites of WipA (grey) and WipB (purple) in the same orientation and representation as in Fig. 2b. Residues in the catalytic sites are shown in ball-and-stick representations. However, only those in different positions when comparing the WipA and WipB structures are labelled, grey and purple for WipA and WipB, respectively. In WipA, Arg123 (185 in WipB), Asp31 (30 in WipB) and Asp331 (396 in WipB) are involved in catalysis while Arg369 of WipA was hypothesized to be involved in pTyr recognition. In WipB, the equivalent residue of Arg369 is Arg310, which is conformationally restrained away from the active site and therefore cannot assume a role in substrate recognition. We hypothesize that this is the reason why WipB is inactive against pTyr-containing peptides (see main text).

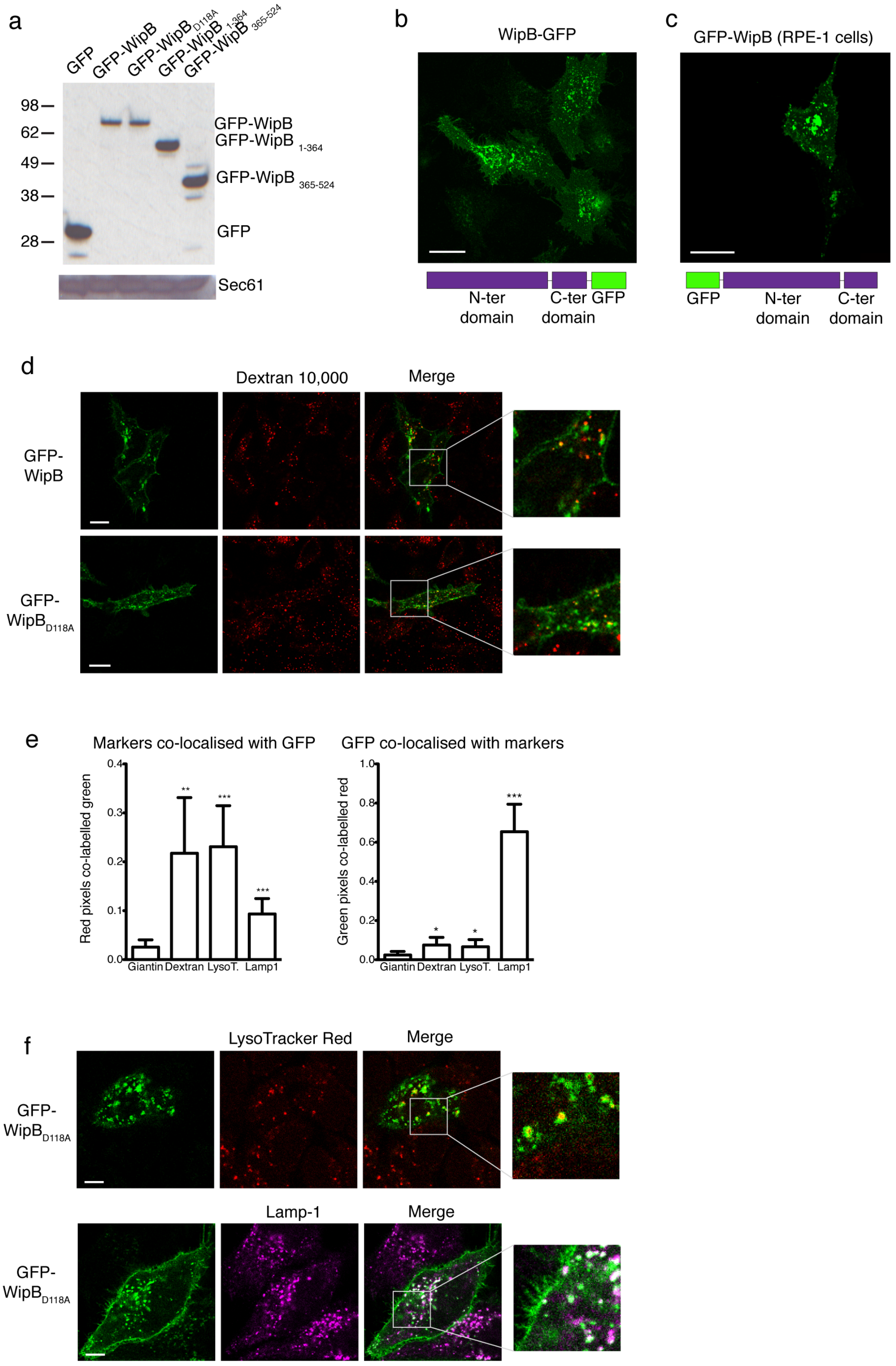

Supplementary Figure 3: Expression of GFP, GFP-WipB and GFP-WipB derivatives in cultured mammalian cells.

a: SDS-PAGE of HeLa cell lysates following transfection with GFP, GFP-WipB or the indicated GFP-WipB derivatives and immunoblotting using an anti-GFP antibody. Eukaryotic Sec61 is included as a loading control.

b: Cultured HeLa cells expressing WipB-GFP (green). Scale bar, 25 µm.
c: Cultured RPE-1 cells expressing WipB-GFP (green). Scale bar, 25 µm.
d: HeLa cells expressing GFP-WipB or GFP-WipB$_{D118A}$ (green) were loaded with TRITC-Dextran 10,000 (red) for 15 before fixation. Scale bar, 10 µm.
e: Quantitative analysis of colocalisation between GFP-WipB and the cellular markers as show in Fig. 3 and Fig. S3, considering the extent of colocalisation of GFP-WipB signal with the markers (left), or conversely the extent of colocalisation of the markers with GFP-WipB (right). *, ** and *** indicate P values < 0.05, < 0.01, < 0.001, respectively, and were calculated employing an unpaired *t*-test.
f: HeLa cells expressing GFP-WipB$_{D118A}$ (green) were loaded with Lysotracker (red) for 15 min before fixation or stained using an anti-LAMP-1 antibody (magenta) after permeabilisation and fixation. Scale bar, 10 µm.

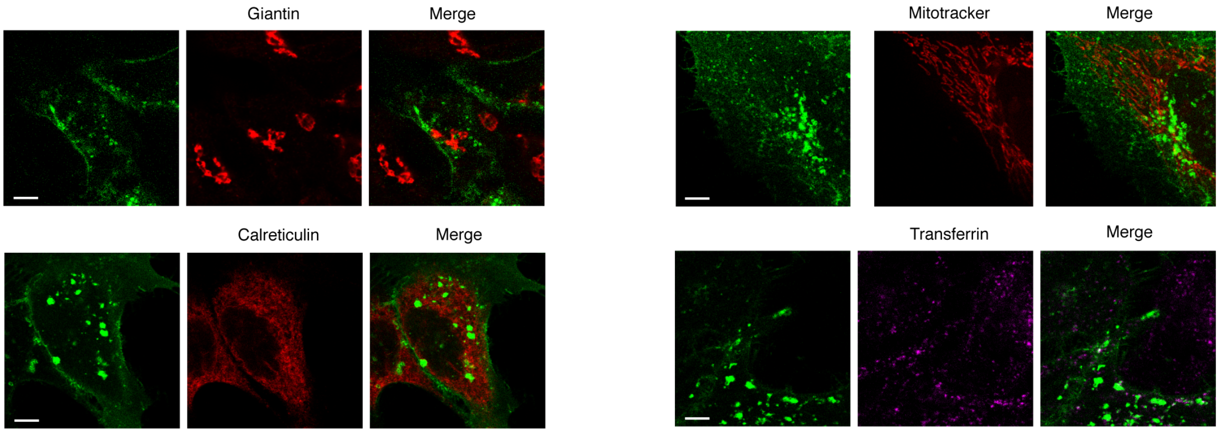

Supplementary Figure 4: Localisation of GFP-WipB in cultured cells

HeLa cells expressing GFP-WipB (green) were fixed and stained with an anti-giantin or anti-calreticulin antibody (red), prior to fixation with Mitotracker (red), or loaded with transferrin (magenta). Scale bars, 10µm.

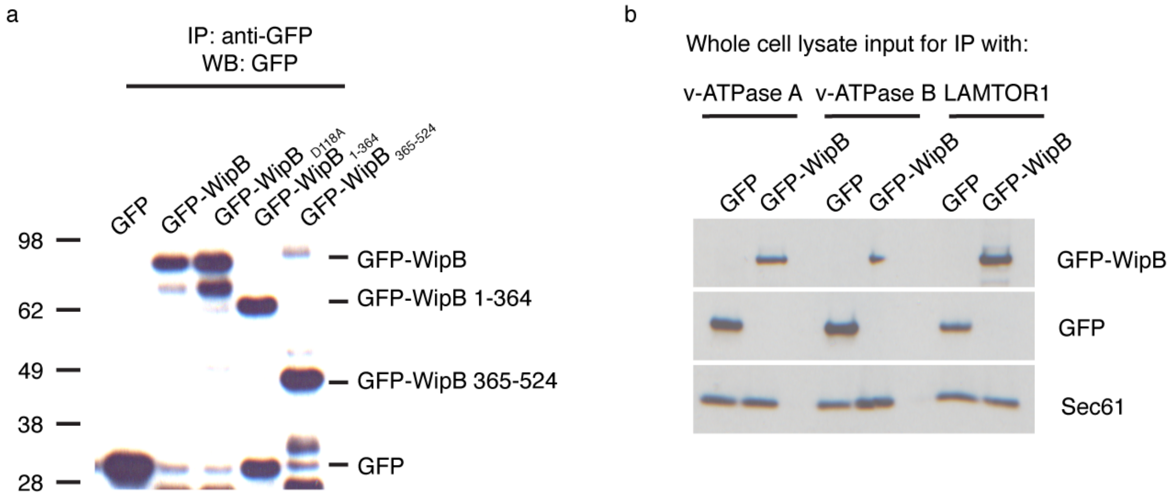

Supplementary Figure 5: Immunoprecipitation of GFP-WipB and LYNUS components

a: SDS-PAGE of HeLa cell lysates (input) following transfection with GFP, GFP-WipB or the indicated GFP-WipB derivatives and immunoblotting with anti-GFP antibody.

b: SDS-PAGE of HeLa cell lysates (input) for the immunoprecipitation experiments using anti-v-ATPase A, anti-v-ATPase B and anti-LAMTOR antibodies shown in Fig. 4E, following immunoblotting with anti-GFP antibody. Identical lysate samples were blotted with anti-Sec61 as a loading control.

References

1. Holm, L. & Rosenström, P. Dali server: conservation mapping in 3D. *Nucleic Acids Res.* **38,** W545–9 (2010).
2. Sievers, F. *et al.* Fast, scalable generation of high-quality protein multiple sequence alignments using Clustal Omega. *Mol Syst Biol* **7,** 539 (2011).
3. Robert, X. & Gouet, P. Deciphering key features in protein structures with the new ENDscript server. *Nucleic Acids Res.* **42,** W320–4 (2014).

Unmodified scans of the gel and blots displayed in the article

Figure 1a

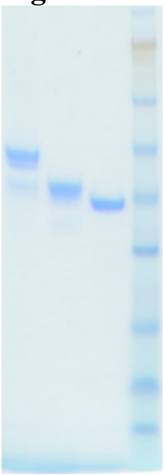

Figure 3d

The membrane was cut in 3 horizontal strips around the molecular weight of the protein of interest (v-ATPase A, 68kDa; v-ATPase B, 57kDa; LAMTOR1, 17kDa). Each strip was incubated with the corresponding primary antibody, then the secondary HRP-conjugated antibody, and finally the ECL reagents. The 3 strips were then re-assembled carefully before exposure.

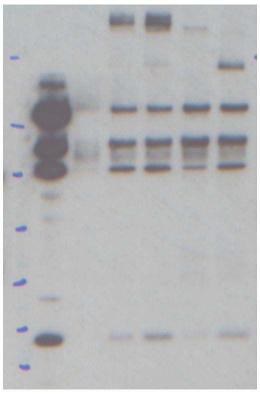

Figure 3e
The membrane was cut in 5 horizontal strips around the molecular weight of the protein of interest (v-ATPase A, 68kDa; v-ATPase B, 57kDa; LAMTOR1, 17kDa; GFP 86kDa and 30kDa). Each strip was incubated with the corresponding primary antibody, then the secondary HRP-conjugated antibody, and finally the ECL reagents. The 5 strips were then re-assembled carefully before exposure.

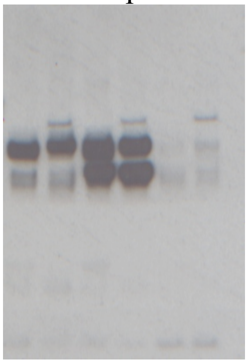

Figure S3a
The membrane was cut in 2 horizontal strips around the molecular weight of the protein of interest (GFP, from 20kDa to top, Sec61 from 20kDa to bottom). Each strip was incubated with the corresponding primary antibody, then the secondary HRP-conjugated antibody, and finally the ECL reagents. The 2 strips were then re-assembled carefully before exposure.

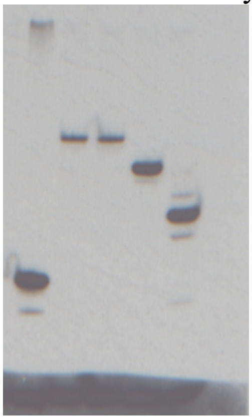

Figure S5a

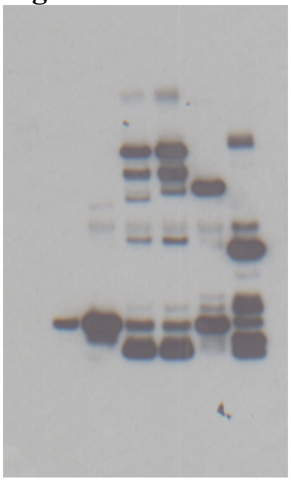

Figure S5b

The membrane was cut in 2 horizontal strips around the molecular weight of the protein of interest (GFP, from 20kDa to top, Sec61 from 20kDa to bottom). Each strip was incubated with the corresponding primary antibody, then the secondary HRP-conjugated antibody, and finally the ECL reagents. The 2 strips were then re-assembled carefully before exposure.

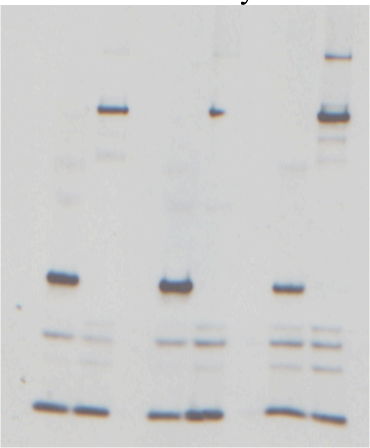

Supplement: Supplementary file 1 — Supplementary information [file 41598_2017_10249_MOESM1_ESM.pdf]
